# Elucidating shared biomarkers and pathways in kidney stones and diabetes: insights into novel therapeutic targets and the role of resveratrol

**DOI:** 10.1186/s12967-023-04356-4

**Published:** 2023-07-21

**Authors:** Shanlin Shen, Jiafeng Wei, Weiting Kang, Tengteng Wang

**Affiliations:** 1 Department of Urology, Affiliated Hospital of Shandong University of Traditional Chinese Medicine, Jinan, 250014 China; 2 Department of Urology, Shandong Provincial Hospital Affiliated to Shandong First Medical University, Jinan, 250021 China

**Keywords:** Kidney stones, Diabetes, Bioinformatics, IL11, Resveratrol, First principles

## Abstract

**Background:**

The pathogenic mechanisms shared between kidney stones and diabetes at the transcriptional level remain elusive, and the molecular mechanisms by which resveratrol exerts its protective effects against these conditions require further investigation.

**Methods:**

To address these gaps in knowledge, we conducted a comprehensive analysis of microarray and RNA-seq datasets to elucidate shared biomarkers and biological pathways involved in the pathogenesis of kidney stones and diabetes. An assortment of bioinformatic approaches was employed to illuminate the common molecular markers and associated pathways, thereby contributing to the identification of innovative therapeutic targets. Further investigation into the molecular mechanisms of resveratrol in preventing these conditions was conducted using molecular docking simulation and first-principles calculations.

**Results:**

The study identified 11 potential target genes associated with kidney stones and diabetes through the intersection of genes from weighted gene co-expression network analysis (WGCNA) and differentially expressed genes (DEGs) screening. Among these, Interleukin 11 (IL11) emerged as a pivotal hub gene and a potential diagnostic biomarker for both conditions, particularly in males. Expression analysis of IL11 demonstrated elevated levels in kidney stones and diabetes groups compared to controls. Additionally, IL11 exhibited correlations with specific cell types and differential expression in normal and pathological conditions. Gene set enrichment analysis (GSEA) highlighted significant disparities in biological processes, pathways, and immune signatures associated with IL11. Moreover, molecular docking simulation of resveratrol towards IL11 and a first-principles investigation of Ca adsorption on the resveratrol surface provided structural evidence for the development of resveratrol-based drugs for these conditions.

**Conclusions:**

Overall, this investigation illuminates the discovery of common molecular mechanisms underlying kidney stones and diabetes, unveils potential diagnostic biomarkers, and elucidates the significance of IL11 in these conditions. It also provides insights into IL11 as a promising therapeutic target and highlights the role of resveratrol. Nonetheless, further research is warranted to enhance our understanding of IL11 targeting mechanisms and address any limitations in the study.

**Supplementary Information:**

The online version contains supplementary material available at 10.1186/s12967-023-04356-4.

## Background

Kidney stones, a persistent and agonizing disorder, is witnessing a rising global prevalence, with an estimated lifetime prevalence of 10% in the general population [1]. This growing trend, coupled with the observed sex gap, has been attributed to factors such as hormonal imbalances, lifestyle habits, and comorbidities, which contribute to abnormal urinary composition and dehydration [1–3]. Nephrolithiasis, rather than being solely a urinary metabolic anomaly, has been shown through research to exhibit connections with multiple metabolic characteristics, encompassing central adiposity, elevated triglyceride levels, hypertension, and diabetes [4]. Diabetes is a significant risk factor for kidney stones, particularly in males, with a 16% higher risk observed in diabetic patients compared to individuals without the condition [5]. Insulin resistance, the hallmark of diabetes, may contribute to reduced urinary pH by disrupting ammoniagenesis and augmenting sodium and bicarbonate reabsorption [6]. Moreover, elevated blood glucose levels and concomitant glycosuria independently heighten urinary calcium excretion, thereby facilitating the development of calcium-based renal calculi in individuals with diabetes [2].

The prevention and treatment of diabetes and kidney stones with medication is a major concern for the general public. While dietary and behavioral interventions alone may be insufficient, the development of safe and effective stone-inhibiting drugs is urgently needed. Resveratrol, a natural polyphenol compound with multiple biological activities, has been shown to help in treating or preventing diabetes and decreasing insulin resistance [7, 8]. Studies have also demonstrated that resveratrol supplementation can curtail the creation of reactive oxygen species triggered by oxalate and diminish the manifestation of profibrotic elements, thereby decreasing kidney stones formation [9, 10].

Nonetheless, the overlapping phenotypic characteristics and molecular signaling pathways underlying the pathogenesis of both kidney stones and diabetes at the transcriptional level continue to be enigmatic. Besides, the mechanisms by which resveratrol can improve diabetes parameters and kidney stones are complex and not yet fully understood [11]. To elucidate the common biomarkers and pathways involved in the mechanism of kidney stones and diabetes, we first identified these biomarkers through microarray and RNA-seq datasets. Various bioinformatics analyses were utilized to unravel the critical biomarkers and biological roles, thereby offering valuable perspectives on potential innovative therapeutic targets. Additionally, we employed the supercomputing platform and quantum mechanical computational theory to analyze the mechanism of resveratrol in preventing renal calcium stones from a new and microscopic perspective, providing basic structural evidence for the development of drugs based on resveratrol.

## Methods

### Data collection

**Fig. 1:**
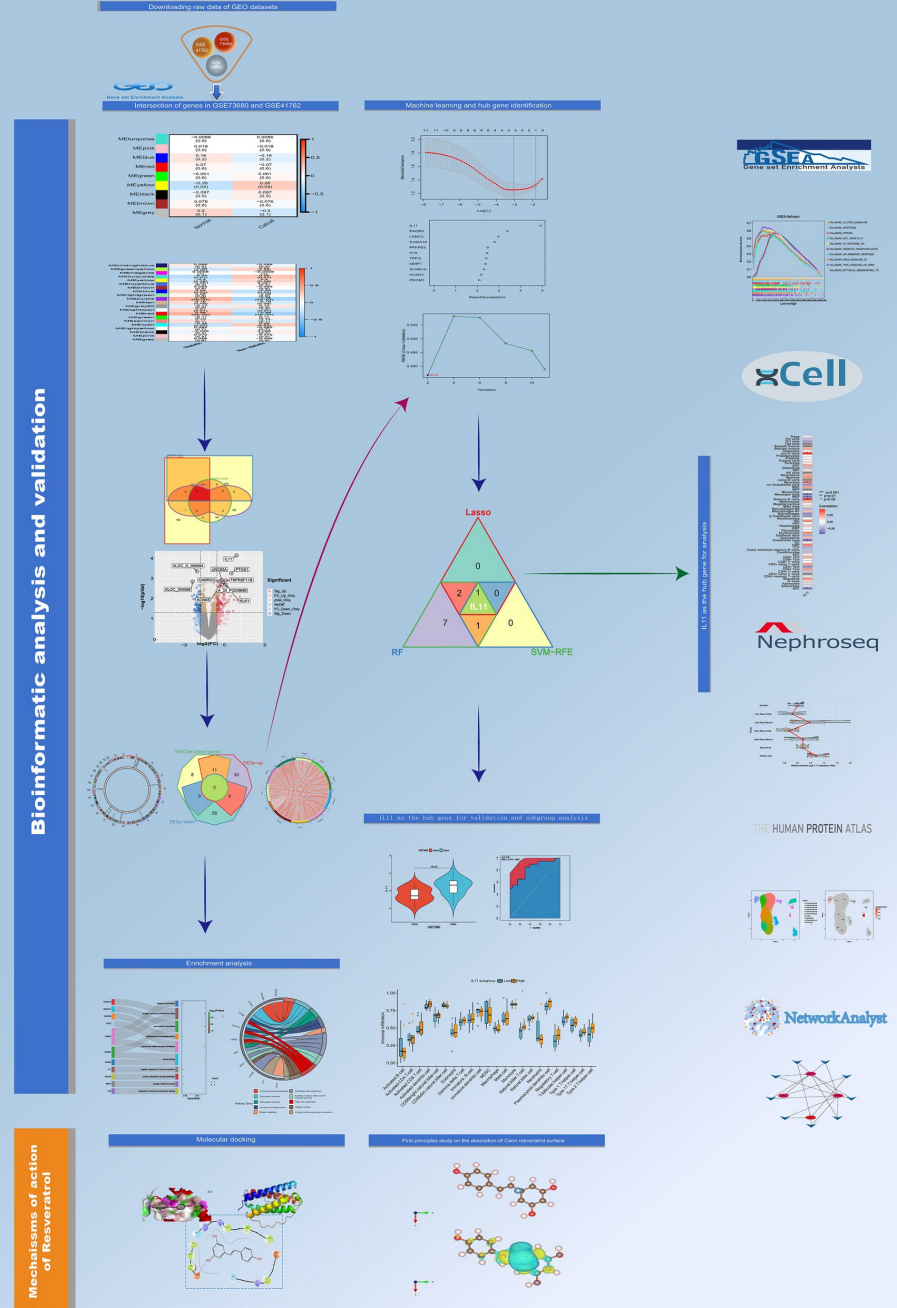
Study flowchart of the whole procedures

A flowchart outlining this study is delineated in Fig. 1. The gene expression omnibus (GEO) database [12], specifically GSE73680, furnished a comprehensive dataset encompassing gene expression profiles from 33 normal renal papillary tissues and 29 tissues afflicted with Randall's plaque (RP). Additionally, GSE41762 (comprising human islet tissues from 57 non-diabetic individuals and 20 individuals with diabetes) and GSE38642 (comprising human islet tissues from 54 non-diabetic individuals and 9 individuals with diabetes) contributed transcriptome data of controls and patients with diabetes.

### Screening of module genes via weighted gene co-expression network analysis (WGCNA)

WGCNA [13] was employed to analyze GSE73680 and GSE41762 datasets to determine gene modules associated with kidney stones and diabetes. Preliminarily, samples underwent evaluation for absent data points before clustering. Following this, a "soft" threshold power (β) was established in accordance with the scale-free topology criterion, facilitating the construction of a biologically significant scale-free network. Furthermore, a topological overlap matrix (TOM) was derived from the adjacency matrix, with a dynamic tree-cutting algorithm employed to identify gene modules. Gene significance (GS), module membership (MM), and modules correlated with clinical characteristics were subsequently computed, culminating in the visualization of the feature gene network. The utilization of the Pearson correlation coefficient and the *P*-value of eigengenes and disease traits facilitated the identification of pivotal modules associated with kidney stones and diabetes. Finally, the significant module genes from WGCNA of kidney stones and diabetes were combined to identify shared genes through AWFE diagrams.

### Identification of differentially expressed genes (DEGs)

The identification of DEGs between the kidney stone cohort and the normal cohort was carried out using the R package "limma", employing criteria of |log2 FC|> 0.5 and *P*-value < 0.05. The results were visualized through volcano plots and a heatmap in R software (version 4.2.2). Subsequently, two sets of genes were generated, one for DEGs in kidney stones and the other for shared gene modules between kidney stones and diabetes, by following the aforementioned steps. A comparison of two sets of genes was conducted to reveal the genes associated with kidney stones and diabetes, and Chow-Ruskey diagrams were constructed. Thereafter, the Circos track plot was employed to map the location of shared genes on chromosomes, and the Circos plot demonstrated the interconnectivity among shared genes. To further investigate the interrelation among the obtained common genes, a network of protein–protein interactions (PPI) was established utilizing GeneMANIA [14].

### Enrichment analysis of shared genes from kidney stones with diabetes

To determine the potential functions among shared genes from kidney stones with diabetes, analysis of Gene Ontology (GO) and Kyoto Encyclopedia of Genes and Genomes (KEGG) pathways was conducted utilizing the "org.Hs.eg.db", "ggplot2", "clusterProfiler", and "enrichplot" packages in the R software. Additionally, Reactome pathway analysis was performed using an online tool that was freely accessible, manually curated and peer-reviewed [15]. Afterward, GO analysis was executed to uncover overlapping gene-associated biological processes (BP), molecular functions (MF), and cellular components (CC). Moreover, KEGG and Reactome analysis were also performed to determine the enriched signaling pathways indicated by the potential targets. Significance of results was established based on the criterion of a *P*-value less than 0.05. Visualizations of the three leading terms of each group were depicted using Sankey plots and chord diagrams, courtesy of the digital arena for conducting data evaluation and depiction.

### Machine learning

To further refine the selection of candidate genes associated with kidney stones in diabetic patients, three machine learning algorithms were employed. Least Absolute Shrinkage and Selection Operator (LASSO), a regression approach, facilitates variable selection to heighten the interpretability and predictive precision of a statistical model [16]. Conversely, Random Forest (RF) offers advantages such as unconstrained variable conditions and superior accuracy, sensitivity, and specificity, making it suitable for the prediction of continuous variables and providing consistent forecasts [17]. Support Vector Machines Recursive Feature Elimination (SVM-RFE) is a sequence selection algorithm based on the maximum interval principle of SVM, wherein all feature sets of the data set are optimized for SVM model training in the first iteration, and then the scores of each feature are calculated in descending order [18]. The feature set with the smallest score is recorded, the feature with the smallest score is deleted, and the iteration is repeated until only one feature remains. Utilizing the R packages "glmnet", "randomForest", "e1071", "kernlab", and "caret" [19–21], we conducted LASSO regression, RF analysis, and SVM-RFE algorithms. The convergence of results obtained from these three machine learning approaches were deemed as potential hub genes in kidney stones with diabetes.

### Hub gene expression and receiver operating characteristic (ROC) evaluation

The hub gene's expression profiles were initially assessed in the GSE73680 and GSE41762 datasets and subsequently verified in the GSE38642 dataset. Moreover, subgroup analyses were performed on the GSE73680 dataset to examine the association between IL11 expression levels and tissue of origin and sex distribution. Specifically, three distinct subcategories were delineated: the "Calculi" subgroup, consisting of Randall's Plaque obtained from calcium stone formers; the "Normal" subgroup, encompassing unaltered papillary tissues samples from calcium stone formers; and the "Control" subgroup, comprising unremarkable papillary tissues derived from patients devoid of any renal calculi. Comparison between these subgroups was performed using the Wilcoxon rank-sum test, with a predetermined significance level of *P* < 0.05. ROC curves were generated to assess the diagnostic merit of the hub gene for kidney stones and diabetic diagnosis, respectively. The AUC values and their corresponding 95% confidence intervals were calculated to distinguish the disease group from the control group, with an AUC value exceeding 0.7 considered to be indicative of a significant difference.

### Evaluating the immune and stromal cell infiltration

To estimate the immune cell composition in kidney stones and control samples, we employed xCell, an online tool that performs cell type enrichment analysis for stromal cell types and 64 immune and applies a novel method to lower confounding effects of closely related cell types [22]. Subsequently, spearman correlation analysis was executed to investigate the correlation between the pivotal genes and diverse cells, and the outcomes were visualized in a heatmap.

### Validation of hub gene expression in nephroseq and HPA database

To ascertain the association between the hub gene and clinical traits, Nephroseq v5 database [23], an integrated webtool for analyzing gene expression datasets related to renal diseases, was employed. Additionally, expression profiles of IL11 and recognized cell type markers in the distinct single cell type clusters of the kidney were procured from the HPA database [24]. Subsequently, a correlation analysis utilizing Pearson’s method was carried out, whereby a statistical significance was assigned to *P* < 0.05.

### Subgroup analysis based on the hub gene expression

To gain a deeper understanding of the hub gene's involvement in kidney stone disease, tissue samples from affected individuals were segregated into low- and high-expression subgroups as per the hub gene's median expression. Gene set enrichment analysis (GSEA) was subsequently applied through the OmicShare tool [25], to distinguish functional and pathway differences between the two subclusters. Volcano plot construction was then performed to pinpoint DEGs with |log2 FC|> 0.5 and *P* < 0.05. Subsequently, the "clusterProfiler" package was utilized for the execution of GO and KEGG analysis, aiming to elucidate the biological roles attributed to the DEGs. Additionally, the Single Sample Gene Set Enrichment Analysis (ssGSEA) was carried out using the "GSVA" R package on 23 immune gene sets to assess the immunological characteristics of the subgroup samples. Lastly, a correlation matrix was constructed for all 23 immunological cell subtypes, along with a correlation coefficient between the hub gene and immune cells with significantly different expression.

### Mfuzz expression pattern clustering of the hub gene

Utilizing the R package “Mfuzz” [26], expression patterns were clustered by considering the hub gene's expression levels. Following this, ssGSEA scores of various clustering modules in the kidney stone group and the normal group were calculated to ascertain the association between the clustering modules and the hub gene. Through this process, we were able to identify the gene module that was most closely associated with the hub gene. We then intersected the genes in this module with the common gene targets of kidney stones and diabetes to derive a set of core genes that are relevant to the hub gene's expression.

### Construction of co-expression network of TF-genes and miRNA-genes

NetworkAnalyst 3.0 [27], a powerful visual analytics tool for conducting in-depth gene expression profiling and meta-analysis, was employed to establish co-expression networks of transcription factors (TFs) and miRNA interacting with the hub gene expression-related genes respectively. A comprehensive set of experimentally validated miRNA-gene interaction data was gathered from the TarBase [28], with TF targets derived from the JASPAR [29].

### Molecular docking validation of resveratrol with the core target

In this study, molecular docking experiments were conducted to investigate the interaction between resveratrol and the core target [30]. Initially, the protein structure that corresponds to the core target was procured from the PDB database. Following this, the Pymol software was employed to eliminate water and ligands from the receptor protein. Additionally, Autodock tools software was utilized for receptor protein modification through hydrogenation and charge balancing. The Grid Box command was employed to access the Grid Option tool, which facilitated the processing of the receptor protein and the determination of the ligand binding pocket dimensions. These dimensions were ascertained based on the lattice points quantity and the inter-point spacing in each direction, with appropriate adjustments made to the lattice points number, binding pocket center, and grid points spacing. To simulate the binding mode of resveratrol with the target protein, Autodock Vina software was employed, and the affinity was subsequently computed to appraise the ligand's binding efficacy to the receptor molecule, with a lower energy value indicating a superior binding effect.

### First-principles investigation of calcium adsorption on resveratrol surface

The comprehensive framework of first-principles methods encompasses all calculations in quantum mechanical principles. These methods provide pertinent insights into the electronic structure properties of a system, facilitating the characterization of chemical bond cleavage, formation, and electronic reorganizations, including chemical reactions. Leveraging their inherent accuracy-enhancement strategies, these theoretical approaches typically require solely the atomic species and coordinates as inputs, enabling precise computation of diverse physicochemical properties exhibited by molecular systems. Currently, first-principles calculations primarily rely on density functional theory (DFT), a method for computing quantum systems that replaces the wave function of the system with a functional of the particle density, thereby avoiding complex calculations in high-dimensional space and facilitating practical and efficient computations [31]. Notably, VASP (Vienna Ab-initio Simulation Package) stands as a well-established software package, crafted by researchers affiliated with the University of Vienna, dedicated to materials calculations predicated on density functional theory [32].

In this study, the adsorption properties of calcium (Ca) on resveratrol (C_14_H_12_O_3_) and oxalic acid (C_2_H_2_O_4_) were investigated via DFT as the first-principles calculation method [31] and the VASP. The generalized gradient approximation (GGA) based Perdew-Burke-Ernzrhof (PBE) functional was used to calculate the exchange–correlation interactions, and the pseudo-potential description method was the projector augmented wave (PAW) method. The K-point was divided into 5 × 5 × 1, and the plane wave cutoff kinetic energy was 500 eV. During the structural optimization stage, the conjugate gradient algorithm was used and the convergence criterion was that the discrepancy in overall energy between two distinct ion stages was below 0.0001 eV. The interaction between them was corrected using the D3 dispersion correction to density functional theory (DFT-D3) grime method. The charge transfer amount during adsorption was analyzed using the Bader charge. The adsorption energy of Ca on the molecular surface, defined as E_ads_, is given by the formula: E_ads_ = E_sub + Ca_ − E_sub_ − E_Ca_. If the calculated E_ads_ is negative, an exothermic reaction occurs when the two interact on the surface, and the more negative the value, the more stable the adsorption structure. The calculation platform was provided by the cluster system of the Shandong University Super Calculation Center.

In this investigation, an analysis of the energy requisite for calcium to adsorb onto C_14_H_12_O_3_ was conducted, initially by ascertaining the structural formula of resveratrol and optimizing the C_14_H_12_O_3_ structure to attain the most stable molecular conformation. Five distinct sites on the resveratrol molecular structure were designated as preliminary positions for computing the adsorption energy of calcium to C_14_H_12_O_3_, with the outcomes compared. Moreover, to further corroborate the stable conformation of the adsorption system, the study delineated the modifications in the C_14_H_12_O_3_ structure following calcium adsorption, and a charge density difference diagram was employed to illustrate the charge distribution shifts pre- and post-calcium adsorption. Ultimately, by establishing a stable adsorption system and calculating the adsorption energy, the investigation compared calcium adsorption on C_14_H_12_O_3_ and C_2_H_2_O_4_.

## Results

### Identifying co-expressed gene modules associated with kidney stones and diabetes

**Fig. 2:**
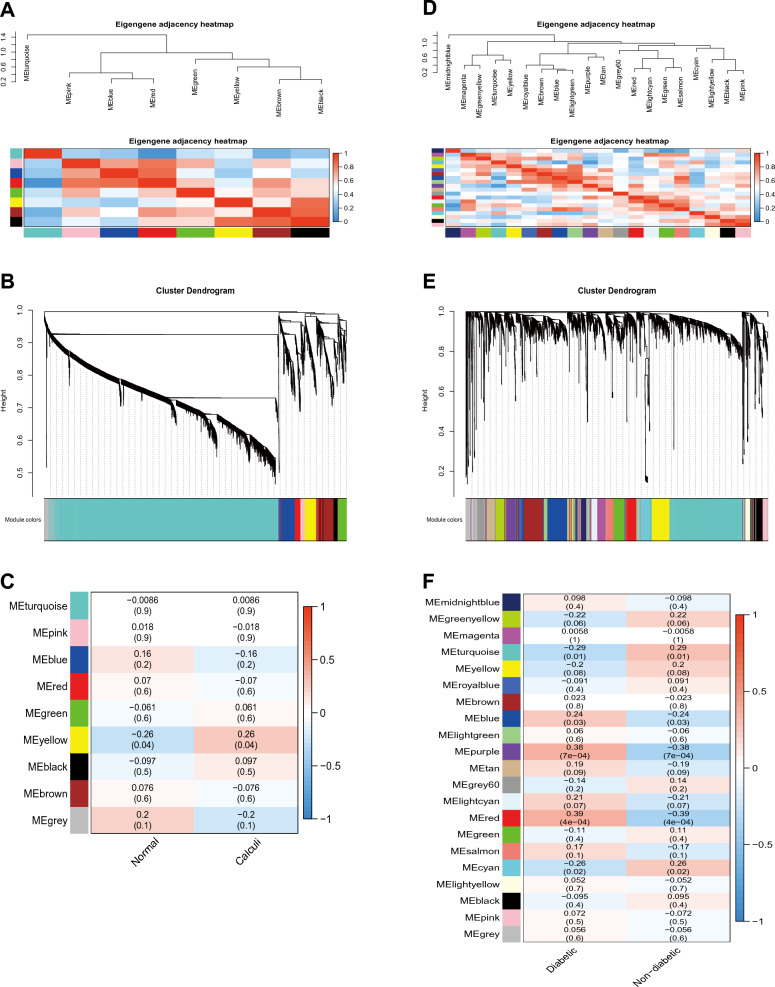
Identification of module genes via WGCNA in the GSE73680 dataset of kidney stones and the GSE41762 dataset of diabetes. **A** Eigengene dendrogram and heatmap to illustrate the meta-modules of correlated eigengenes for the GSE73680 dataset of kidney stones. **B** Clustering dendrogram and merging of the gene co-expression modules represented by different colors in kidney stones. **C** Heatmap of the module–trait relationship in kidney stones. At the row column intersection lies the correlation and p-value that correspond to each other. **D** Eigengene dendrogram and heatmap to illustrate the meta-modules of correlated eigengenes for the GSE41762 dataset of diabetic. **E** Clustering dendrogram and merging of the gene co-expression modules distinguished by varying colors in diabetic. **F** Heatmap of the module–trait relationship in diabetic. At the row column intersection lies the correlation and p-value that correspond to each other

As shown in Fig. 2A–C, the WGCNA analysis of GSE73680 dataset, comprising of 33 normal papillary tissues and 29 RP papillary tissues, resulted in the identification of 9 gene modules associated with kidney stones. No samples were found to be outliers (Additional file 1: Fig. S1A). The connection linking each module and the disease was appraised through a heatmap and the Spearman's correlation coefficient. The MEyellow module was found to be positively correlated with kidney stones (r = 0.26, *P* = 0.04) and was therefore identified as the pivotal module for further scrutiny. Similarly, as shown in Fig. 2D–F, the WGCNA analysis of GSE41762 dataset, which included human islet tissues from 57 normal and 20 diabetic groups, identified 21 gene modules associated with diabetic. No samples were removed as outliers (Additional file 1: Fig. S1B). The MEblue, MEpurple and MEbrown modules were found to be positively correlated with diabetes (r = 0.24,* P* = 0.03; r = 0.38,* P* = 7e−04; r = 0.39,* P* = 4e−04) and were selected as key modules for further analysis, with 437, 160, and 183 genes, respectively.

### Determination of DEGs in the GSE73680 dataset of kidney stones

**Fig. 3:**
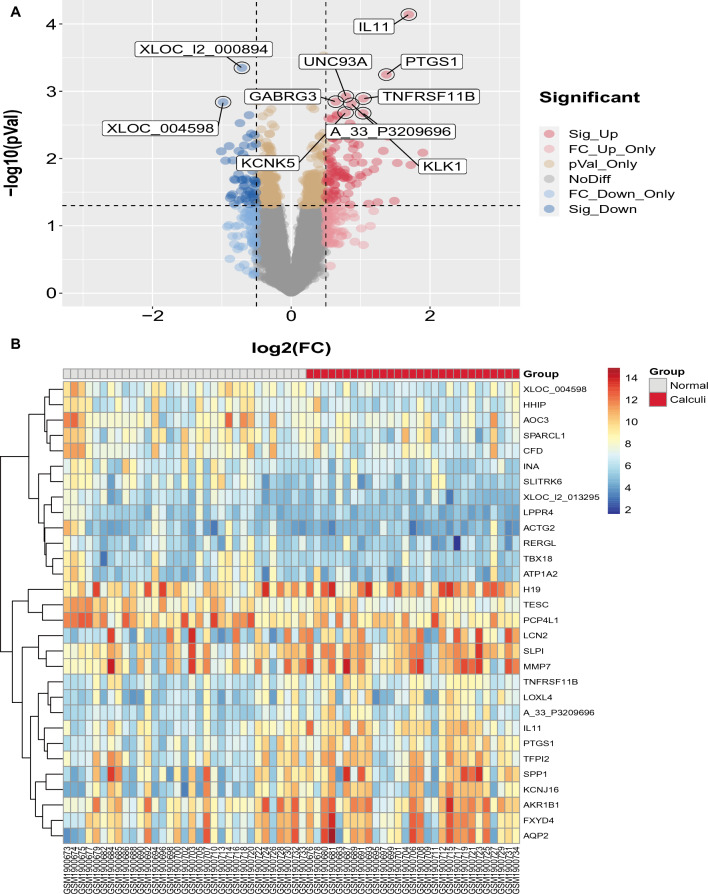
Identification of DEGs in the GSE73680 dataset of kidney stones. **A** Volcano plots showing all DEGs. **B** Heatmap to visualize the top 30 DEGs

Through rigorous analysis, a comprehensive list of 151 DEGs were generated, comprising 58 downregulated genes and 93 upregulated genes, as evidenced in the volcano plot (Fig. 3A). Additionally, to delve deeper into the most prominent differences between the disease and control cohorts, a heatmap was generated (Fig. 3B) showcasing the top 30 genes exhibiting the the greatest degree of variation.

### Enrichment analysis of shared genes from kidney stones and diabetes

**Fig. 4:**
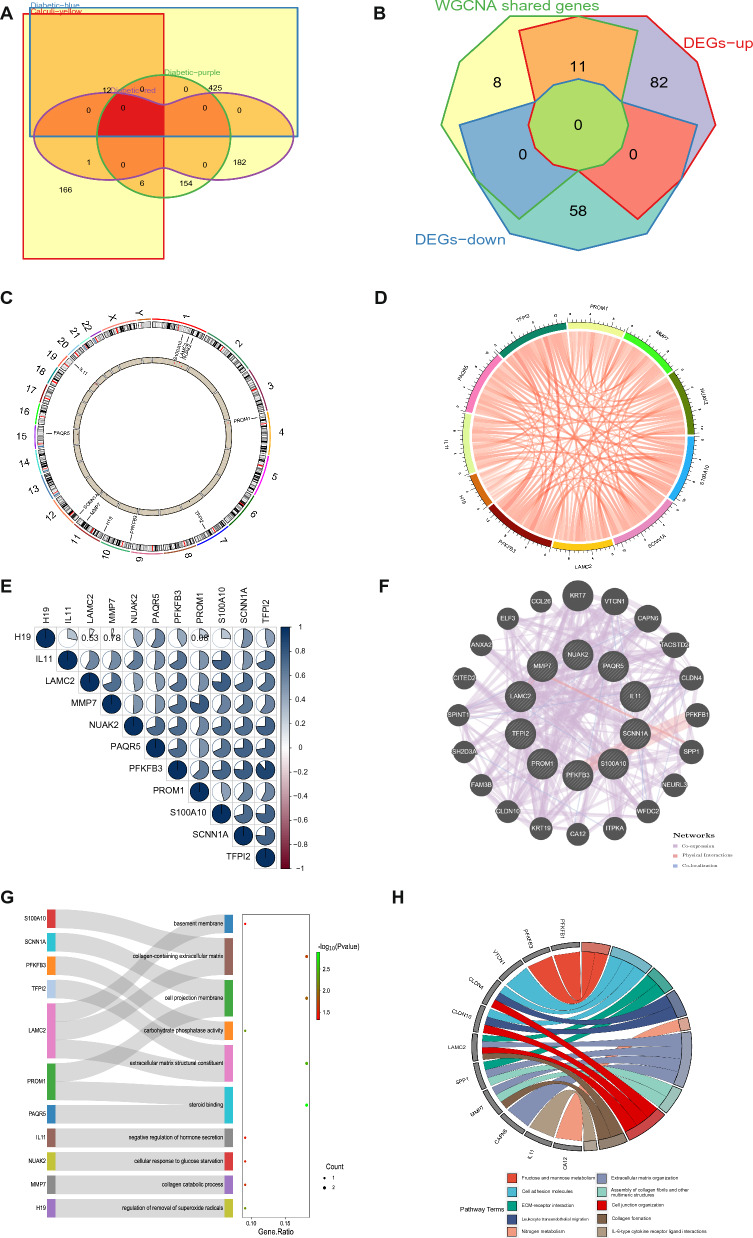
Enrichment analysis of shared genes from kidney stones with diabetes. **A** AWFE sets of the shared genes between the MEyellow module of kidney stones and MEturquoise, MEblue, MEpurple, MEred, MEcyan modules of diabetic by overlapping them. **B** Chow-Ruskey diagrams of 17 common genes identified from the intersection of DEGs in kindey stones using Limma and the shared genes between kidney stones and diabetic using WGCNA. Color of the borders around each intersection corresponds to the modules whose genes overlap. The green circle in the middle represents the overlap of all modules. Lighter shades of brown, blue and pink represent the overlap of fewer modules. Area of each intersection is proportional to number of genes within the intersection. **C** Circos track plot used to map the location of 11 shared genes on the chromosomes. **D** Circos plot demonstrating the interconnectivity among 11 shared genes. The hue and width of the ribbons are associated with the expression levels of genes, with red signifying a positive relationship and green indicating a negative one. **E** Matrix graphs of correlation analysis of 11 shared genes. **F** PPI network for the obtained 11 common genes constructed by GeneMANIA. **G** GO enrichment analysis of the obtained 11 common genes. **H** KEGG and Reactome enrichment analysis of the obtained 11 common genes

The intersection of genes associated with kidney stones and diabetes was identified by comparing the genes present in positively related modules for both conditions. Using AWFE diagrams, a total of 19 common genes were found across the four relevant modules in kidney stones and diabetes (Fig. 4A). Further analysis utilizing WGCNA-derived significant module genes and DEGs yielded 11 potential target genes for both diseases through Chow-Ruskey diagrams (Fig. 4B). The Circos track plot was used to visualize the location of these shared genes on chromosomes and provided insight into potential regions of genetic overlap between the two diseases (Fig. 4C). The shared genes were discovered to have a positive correlation in their expression levels, as demonstrated by the GEO data in the Circos plot (Fig. 4D). Correlations among the shared genes were also examined using a coloring scheme, with blue signifying positive correlation and red signifying negative correlation (Fig. 4E). A numerical expression was used when the *P*-value was 0.05 or greater. The PPI network for the candidate genes was created via the GeneMANIA database (Fig. 4F), and functional analysis was conducted using GO, KEGG, and Reactome to identify potential mechanisms of action. The results revealed GO enrichment in regulation of regulation of removal of superoxide radicals, collagen catabolic process, cellular response to glucose starvation, negative regulation of hormone secretion (BP); steroid binding, extracellular matrix structural constituent, carbohydrate phosphatase activity (MF); and cell projection membrane, collagen-containing extracellular matrix (ECM), basement membrane (CC) (Fig. 4G). Additionally, the genes exhibited a significant enrichment in KEGG pathways including fructose and mannose metabolism, cell adhesion molecules, ECM-receptor interaction, leukocyte transendothelial migration, AMPK signaling pathway, and nitrogen metabolism (Fig. 4H). Reactome analysis also demonstrated that the targets were intricately linked with ECM organization, assembly of collagen fibrils and other multimeric structures, cell junction organization, collagen formation, and IL-6-type cytokine receptor ligand interactions (Fig. 4H).

### Screening for candidate diagnostic biomarkers for kidney stones in individuals with diabetes using machine learning algorithms

**Fig. 5:**
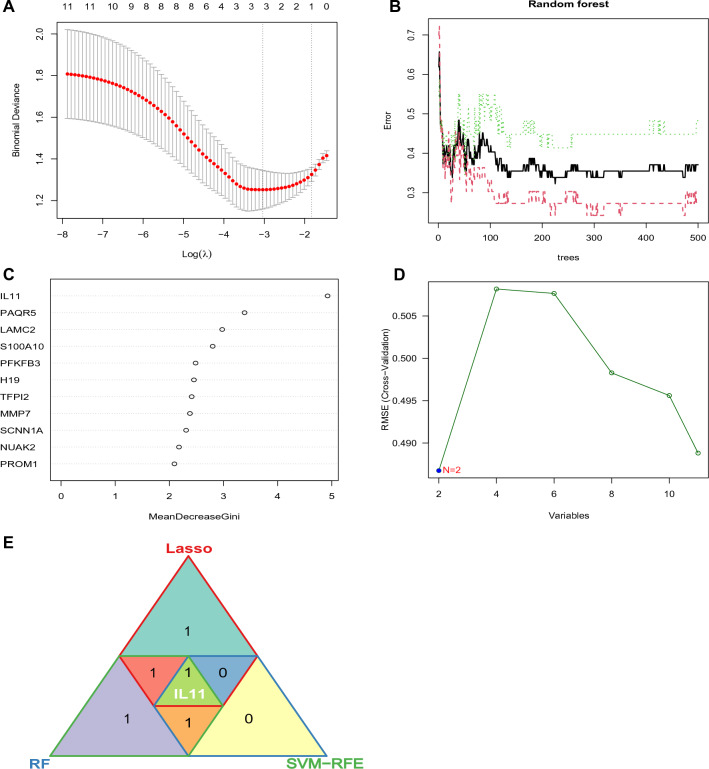
Screening for candidate diagnostic biomarkers for kidney stones with diabetic using machine learnings. **A** Screening for biomarkers using the Lasso model. 3 genes corresponding to the lowest point of the regression curve are the most suitable candidates for kidney stones with diabetic diagnosis. **B** The error in kidney stones shown by random survival forests algorithm. **C** Importance of Genes ranked by MeanDecreaseGini value. **D** Screening for biomarkers using the SVM-RFE algorithm. **E** Triangle venn diagram showing the genes shared/unique among the above three algorithms

Various machine learning algorithms were employed to identify potential diagnostic biomarkers from a set of 11 shared genes identified in both kidney stones and diabetes. The LASSO regression algorithm identified three biomarkers as demonstrated in Fig. 5A. By contrast, the RF algorithm determined four potential biomarkers based on their importance, depicted in Fig. 5B, C. Moreover, the SVM-RFE analysis showed that a model involving two genes achieved the highest accuracy, as illustrated in Fig. 5D. By comparing the results of these three algorithms, a single potential biomarker, IL11, was identified as a shared biomarker for both kidney stones and diabetes (as demonstrated in Fig. 5E).

### Expression characteristics and diagnostic capability assessment of the candidate diagnostic biomarker

**Fig. 6:**
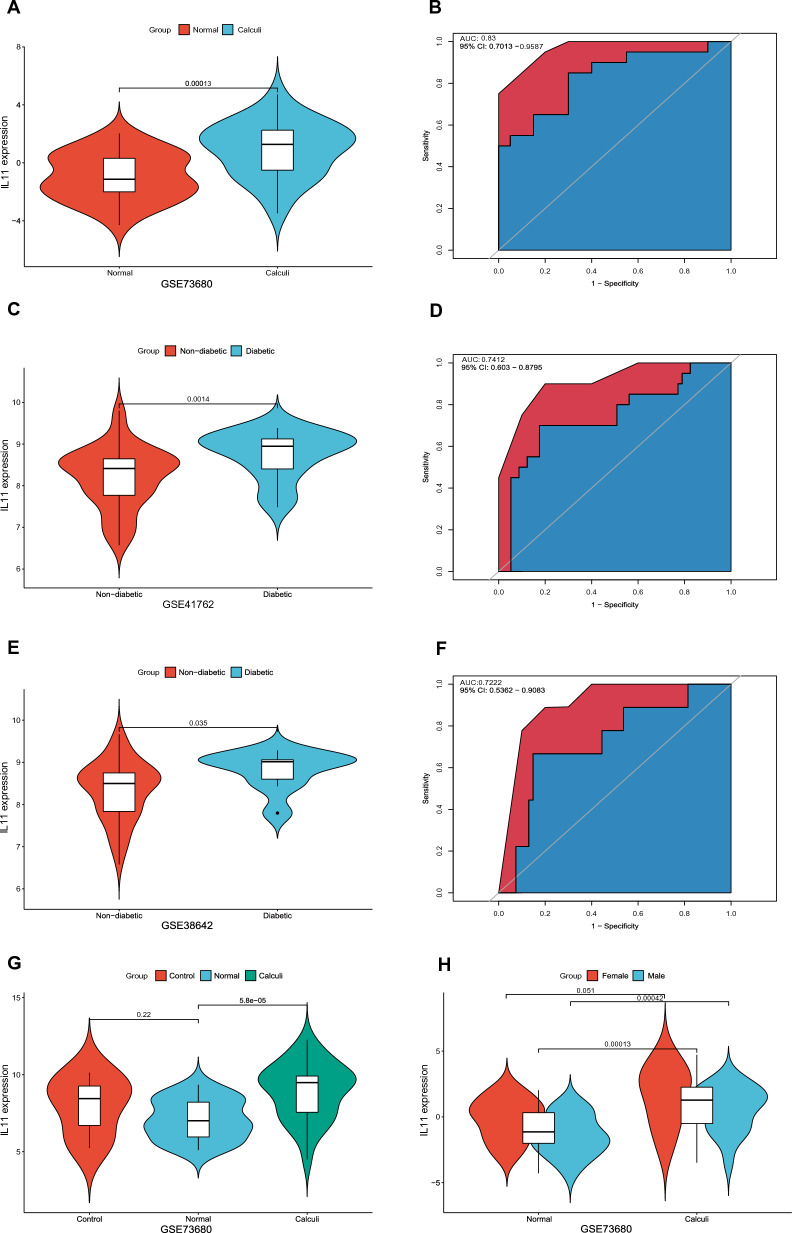
Verification of IL11 as the hub gene. **A** The expression level of IL11 in the GSE73680 dataset of kidney stones. **B** ROC curve for IL11 in the GSE73680 dataset of kidney stones. **C** The expression level of IL11 in the GSE41762 dataset of diabetic. **D** ROC curve for IL11 in the GSE41762 dataset of diabetic. **E** The expression level of IL11 in the GSE38642 dataset of diabetic. **F** ROC curve for IL11 in the GSE38642 dataset of diabetic. **G** Subgroup analysis of IL11 expression levels based on tissue of origin. **H** Subgroup analysis of IL11 expression levels in kidney stones according to sex distribution in the GSE73680 dataset The Wilcoxon rank-sum was employed to compare the two data sets, and a P-value below 0.05 was considered statistically significant

This investigation sought to expand on the significance of IL11 in the onset of nephrolithiasis among individuals afflicted with diabetes. To this end, our approach involved a comprehensive analysis of the expression profiles of IL11 in a patient cohort and compared it with that of a control group. Our results indicated a marked elevation in the levels of IL11 expression in both kidney stones group (as illustrated in Fig. 6A) and diabetes group (Fig. 6C) when contrasted with the control cohort. Despite the absence of another appropriate database to validate IL11 expression levels in kidney stones, our findings were confirmed by analysis of the GSE38642 dataset of diabetes patients (Fig. 6E). The diagnostic potential of IL11 was further evaluated using ROC curves based on the GSE73680 and GSE41762 datasets, which suggest that IL11 could potentially act as a diagnostic biomarker for kidney stones in diabetic patients (Fig. 6B, D). The ROC analysis of the GSE38642 dataset further reinforced the potential of IL11 as the most promising diagnostic marker for this condition (Fig. 6F). Additionally, subgroup analysis of IL11 expression levels based on tissue of origin indicated a significant upregulation of IL11 expression in the "Calculi" group when contrasted with the "Normal" group, but "Normal" and the "Control" groups exhibited no substantial differentiation. (Fig. 6G). Moreover, we investigated the distribution of IL11 expression levels by sex and found a substantial elevation in male patients, but no significant difference was observed in female patients (Fig. 6H). These results provide compelling evidence for the potential of the IL11 signature as an excellent diagnostic biomarker for kidney stones in individuals with diabetes.

### IL11 as the hub gene used for cell type enrichment analysis and expression analysis in Nephroseq and HPA database

**Fig. 7:**
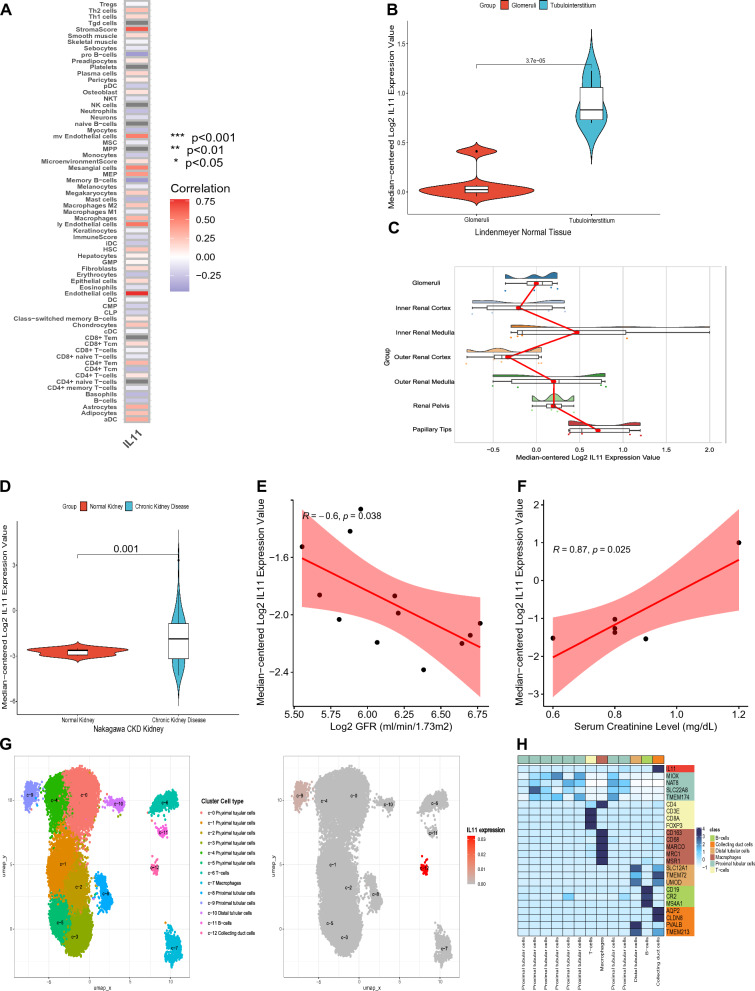
IL11 as the hub gene used for cell type enrichment analysis and expression analysis. **A** Correlation analysis among IL11 and 64 immune and stroma cell types in renal papillary Randall’s plaque (RP) and normal papillary tissue. **B** IL11 over-expression in tubulointerstitium vs. glomeruli tissue of the normal adult human kidney explored in the Nephroseq database. **C** IL11 over-expression in papillary tips vs. all other tissues of the normal adult human kidney explored in the Nephroseq database. **D** IL11 over-expression in chronic kidney disease vs. normal kidney explored in the Nephroseq database. **E** IL11 expression correlated with GFR in healthy living donors explored in the –Nephroseq database. **F** IL11 expression correlated with serum creatinine in open donor nephrectomy samples explored in the Nephroseq database. **G** Expression profiles of IL11 in kidney cell types based on the HPA database. **H** Heatmap for the expression of IL11 and recognized cell type markers across distinct single cell type clusters of the kidney based on HPA database

An xCell enrichment analysis was performed on gene expression data from 64 immune and stroma cell types, revealing a strong positive correlation between IL11 and several cell types including pro B-cells, mv Endothelial cells, Mesangial cells, Memory B-cells, ly Endothelial cells, and Endothelial cells (Fig. 7A). This analysis provides a comprehensive depiction of the cellular heterogeneity landscape in tissue expression profiles. Further examination of IL11 expression levels in various databases showed notable differences between normal and pathological conditions. In the Lindenmeyer Normal Tissue Panel in Nephroseq v5 database, higher expression of IL11 was observed in the tubulointerstitium compared to the glomeruli (Fig. 7B). In the Higgins Normal Tissue Panel, IL11 was over-expressed in papillary tips compared to other structures in the kidney (Fig. 7C). IL11 expression was found to be over-expressed in chronic kidney disease with tubulointerstitial fibrosis and tubular cell damage, compared to normal kidney, in the Nakagawa CKD Kidney in Nephroseq v5 database (Fig. 7D). Analysis of the Woroniecka Diabetes in Nephroseq v5 database showed a significant negative correlation between IL11 expression and the value of GFR (Fig. 7E). Additionally, in the Kurian Transplant Kidney in Nephroseq v5 database, IL11 expression was found to be significantly positively correlated with serum creatinine levels in open donor nephrectomy samples (Fig. 7F). Through single-cell analysis in the HPA database, IL11 was predominantly enriched in kidney collect duct cells (Fig. 7G, H). These results provided important insights into the expression and distribution of IL11 in normal and pathological conditions, offering valuable information for further investigation into its role in renal biology.

### GSEA of biological functions and pathways based on IL11 expression

**Fig. 8:**
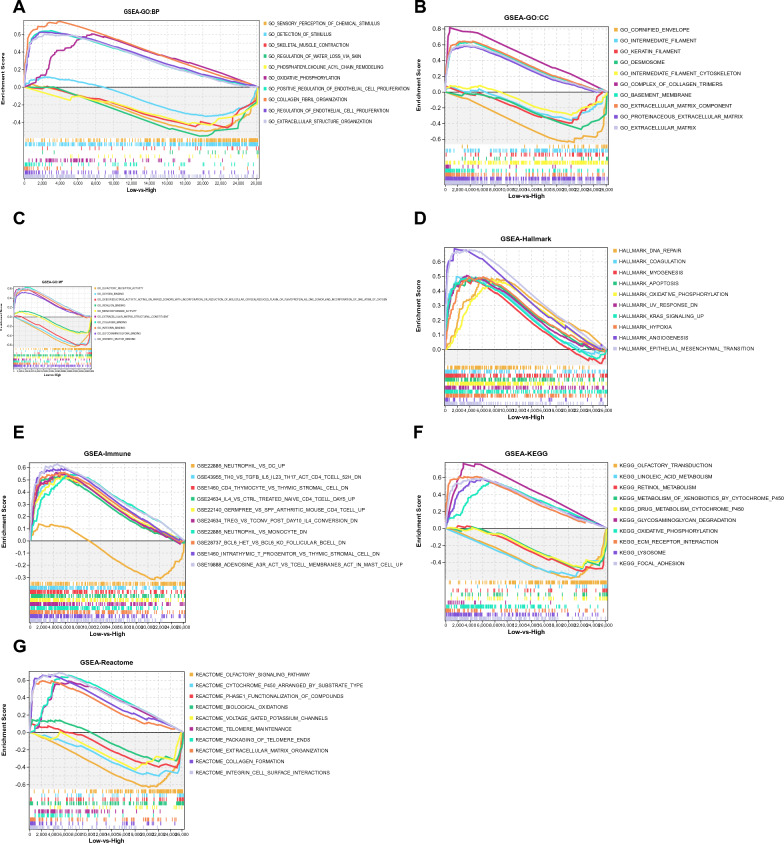
GSEA between kidney stones subtypes based on IL11 expression levels. **A** GO:BP analysis via GSEA. **B** GO:CC analysis via GSEA. **C** GO:MF analysis via GSEA. **D** KEGG pathway analysis via GSEA. **E** Hallmark pathway analysis via GSEA. **F** Reactome pathway analysis via GSEA. **G** Enriched immune cell types via GSEA

The male RP papillary tissues were categorized into two subgroups, low-IL11 and high-IL11, by considering the median expression level of IL11. GSEA was conducted to ascertain biological activities and pathways with differential expression. The results of the GO analysis revealed significant differences in BP, CC, and MF between these two subgroups. The low-IL11 subgroup was enriched in extracellular structure organization, regulation of endothelial cell proliferation, collagen fibril organization, and oxidative phosphorylation, while the high-IL11 subgroup exhibited higher levels of phosphatidylcholine acyl chain remodeling and sensory perception of chemical stimulus (Fig. 8A). In terms of cellular components, the low-IL11 subgroup displayed increased enrichment in the extracellular matrix and basement membrane, whereas the high-IL11 subgroup exhibited higher levels of intermediate filament cytoskeleton (Fig. 8B). Similarly, at the molecular function level, the low-IL11 subgroup showed higher enrichment in growth factor binding and extracellular matrix structural constituents, while the group categorized as high-IL11 displayed elevated levels of both monooxygenase activity and olfactory receptor activity (Fig. 8C). Furthermore, GSEA revealed significant differences in hallmark activities of epithelial mesenchymal transition, angiogenesis, hypoxia, KRAS signaling, oxidative phosphorylation, apoptosis, myogenesis, coagulation, and DNA repair between the low-IL11 and high-IL11 subgroups (Fig. 8D). The analysis of immune signatures also showed differences between the two subgroups, with elevated levels of certain signatures in the low-IL11 subgroup and decreased levels of one signature in the high-IL11 subgroup (Fig. 8E). KEGG and Reactome-based pathway analyses demonstrated differential enrichment of pathways between the two subgroups, highlighting the role of IL11 in pathways related to ECM receptor interaction, focal adhesion, integrin cell surface interactions, collagen formation, and voltage gated potassium channels (Fig. 8F, G).

### Identification of DEGs, functional distinctions and immune cell infiltration between the kidney stones subtypes

**Fig. 9:**
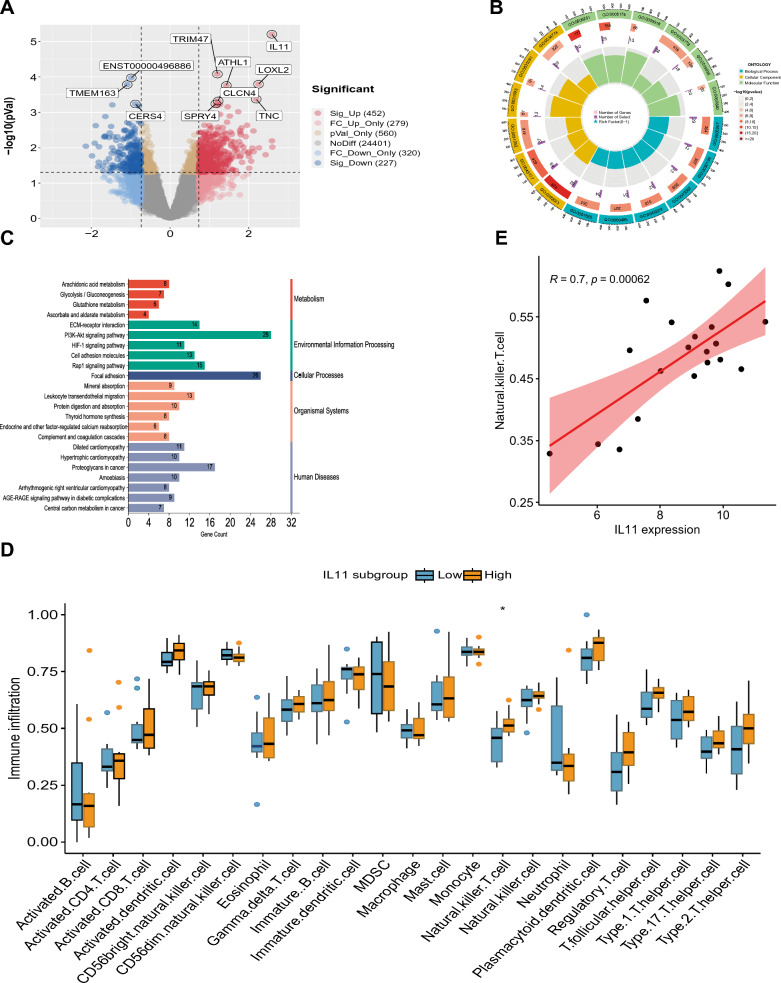
Identification of DEGs, Functional enrichment analysis and Immune cell infiltration between kidney stones subtypes. **A** Volcano plots showing all DEGs. **B** Enriched GO items. **C** Enriched KEGG items. **D** Correlation matrix for all 23 immunological cell subtypes. **E** Correlation coefficient between IL11 and Natural killer T cell

To elucidate the functional discrepancies between the subgroups of kidney stones with low and high levels of IL11, an analysis of DEGs was executed, leading to the discovery of 679 genes comprising 452 upregulated and 227 downregulated genes which were visualized on a volcano plot (Fig. 9A). Subsequent scrutiny was performed via GO and KEGG analysis to investigate the potential molecular processes and functions. GO analysis indicated enrichment in various biological processes, including heart morphogenesis, extracellular matrix organization, mesenchyme development, and urogenital system development (BP). It also revealed enrichment in cellular components including the collagen-containing extracellular matrix and platelet alpha granule (CC), as well as molecular functions such as integrin binding, collagen binding, and actin binding (MF) (Fig. 9B). KEGG pathway analysis indicated enrichment in several pathways related to metabolism, environmental information processing, organismal systems, cellular processes, and human diseases, as presented in Fig. 9C. Additionally, GSVA revealed significantly higher levels of Natural Killer T (NKT) cells in patients in the IL11-high subgroup (Fig. 9D), with a direct relationship between the manifestation of IL11 and NKT cells (Fig. 9E). These findings provide a comprehensive understanding of the functional differences between the two subgroups and could potentially inform future studies.

### Identification and TF-miRNA interaction network analysis of IL11 expression-related genes

**Fig. 10:**
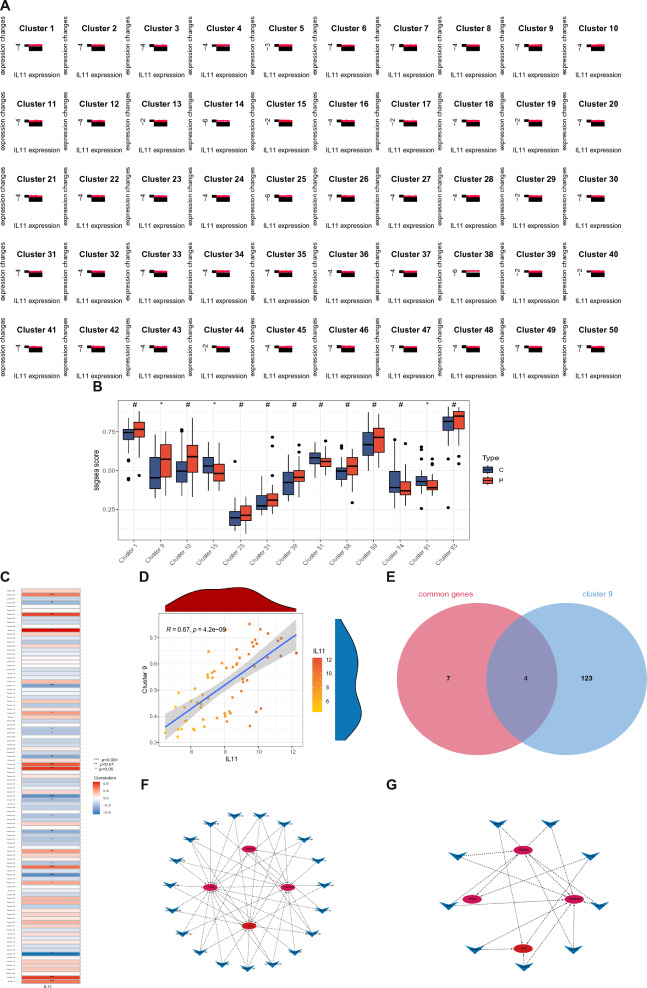
Identification and TF-miRNA interaction network analysis of IL11 expression-related genes. **A** IL11 expression patterns identified by MFuzz. **B** The score of clustering module and expression characteristics comparing normal and calculi groups by ssGSEA. **C** Correlation between clustered modules and IL11. **D** Correlation between cluster 9 and IL11. **E** Venn diagram of common genes and cluster 9 identifying core genes related to IL11 c expression in diabetic with kidney stone. **F** Co-expression network of TF interacting with IL11 expression-related genes. **G** Co-expression network of miRNA interacting with IL11 expression-related genes

In this research, we performed an analysis of IL11 expression-related genes using Mfuzz followed by grouping the results into 100 clusters. The first 50 clusters were visually represented in Fig. 10A, while the other 50 were provided as Additional file 2: Fig. S2. Using a combination of ssGSEA scores and expression attributes, the clustering modules between the kidney stones cohort and the normal cohort were compared, as depicted in Fig. 10B. The correlation between the class modules and IL11 is presented in Fig. 10C, where it was found that Cluster 9 exhibited the strongest association to IL11, as shown in Fig. 10D. Through an intersection of common genes from kidney stones with diabetes and the Cluster 9 module genes, we identified PROM1, TFPI2, and PFKFB3 as core genes related to IL11 expression in kidney stones (Fig. 10E). To uncover the regulatory mechanisms of IL11, PROM1, TFPI2, and PFKFB3, a network of miRNAs and IL11-related genes was constructed (Fig. 10F). Furthermore, potential TFs were predicted and a network of TFs and potential biomarkers was established (Fig. 10G). Ultimately, our findings identified 19 miRNAs and 9 TFs involved in the regulation of IL11.

### Visualization of molecular docking simulation of resveratrol towards IL11

**Fig. 11:**
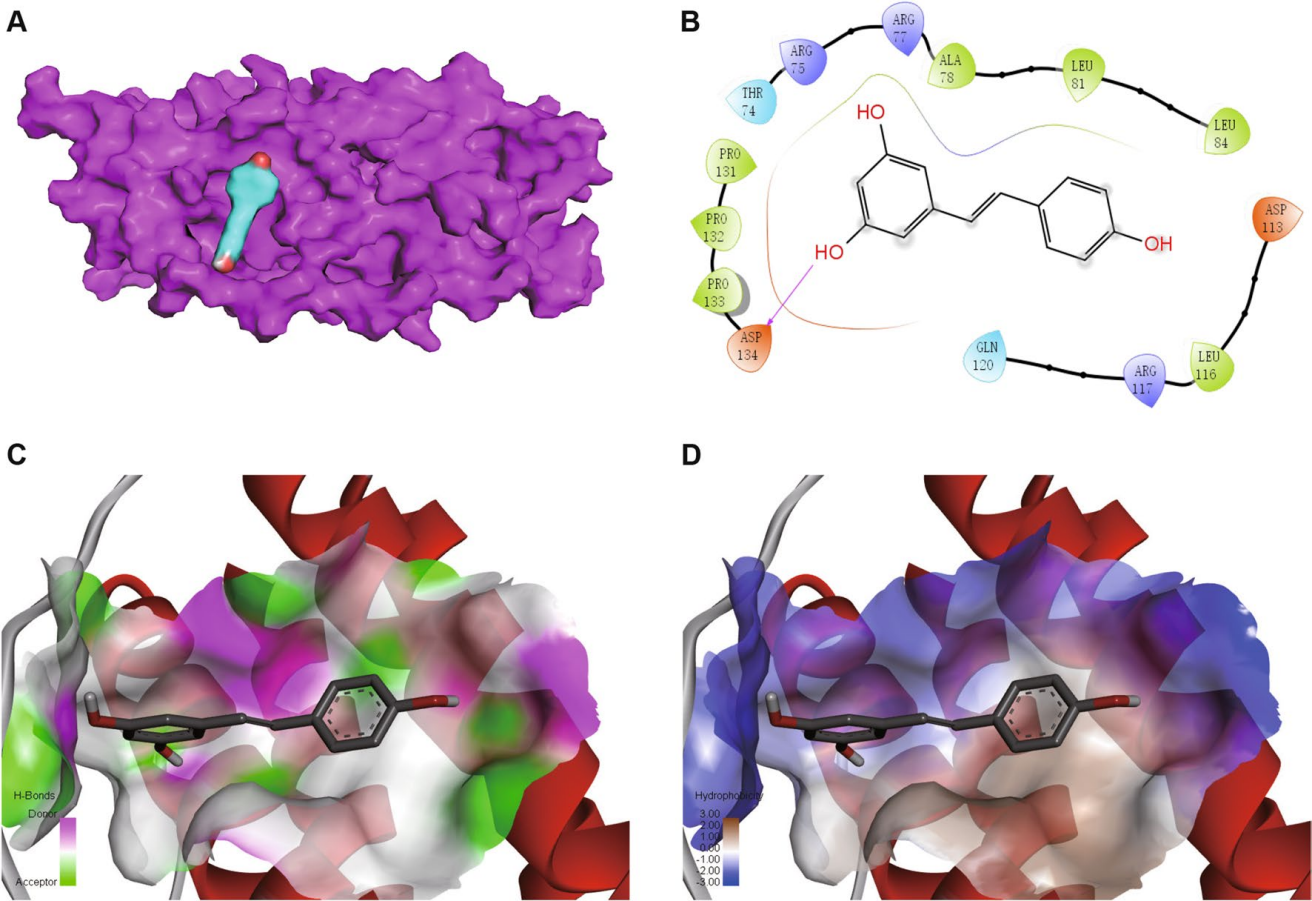
Visualization of molecular docking simulation of resveratrol towards IL11. **A** Docking models for resveratrol-IL11. Resveratrol and IL11 subunit are illustrated in stick-form and as cartoons respectively. **B** 2D molecular interactions are illustrated for resveratrol-IL11. The pyrazole nitrogen of resveratrol forms a hydrogen bond with ASP134 of IL11. The interactions involving hydrogen bonds are depicted through green dashed lines, while van der Waals interactions are represented by light green dashed lines. The alkyl interaction is denoted by a pink dashed line. The docking analyses were performed using BIOVIA Discovery Studio, with details provided in the Methods Sect. 3D molecular interactions are illustrated for** C** hydrogen bonds between resveratrol and IL11 within the binding site, and **D** hydrophobicity surface view at the interface between resveratrol and IL11, respectively. The depiction of resveratrol's molecular structure is rendered in bold stick form, with potential binding sites being indicated by light stick forms

As depicted in Fig. 11A, the molecular structures of resveratrol and IL11 subunit were illustrated in stick form and cartoon representation, respectively. In Fig. 11B, the 2D molecular interactions between resveratrol and IL11 were depicted, highlighting the creation of a hydrogen bond between ASP134 of IL11 and the pyrazole nitrogen of resveratrol. The hydrogen-bond interactions were indicated by green dotted lines, while light green and pink dotted lines represent van der Waals and alkyl interactions, respectively. The docking analyses were performed using BIOVIA Discovery Studio, as described in the Methods section. The 3D molecular interactions between resveratrol and IL11 were illustrated in Fig. 11C, D, displaying the hydrogen bonds within the binding site (Fig. 11C) and the hydrophobicity surface view at the interface between resveratrol and IL11 (Fig. 11D). In these representations, resveratrol was shown in bold stick form, and potential binding sites were indicated in light stick form. The outcomes of the analysis demonstrate a strong binding ability between IL11 and resveratrol, with a binding strength of − 6.8 kcal/mol, as outlined in Table 1.

**Table 1:** Molecular docking sites and energies between resveratrol and IL11

Target protein (PDB ID)	Drug	Amino acid residues of the binding site	Binding affinity (kcal/mol)
IL11(6O4O)	Resveratrol	ASP134, PRO133, PRO132, PRO131, THR74, ARG75, ARG77, ALA78, LEU81, LEU84, ASP113, LEU116, ARG117, GLN120	− 6.8

### First-principles investigation of Ca adsorption on the resveratrol surface

**Fig. 12:**
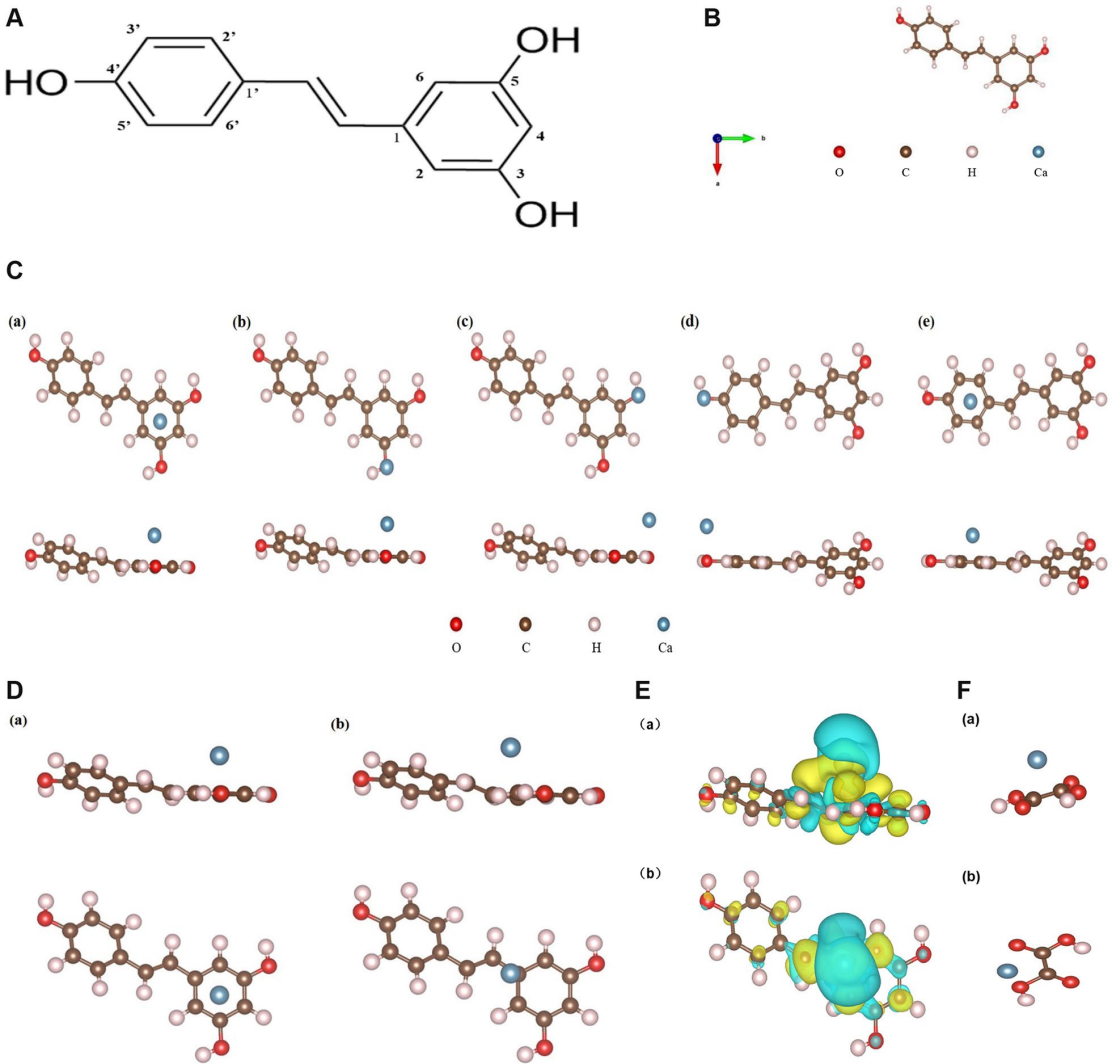
First principles study on the adsorption of Ca on resveratrol surface. The structural formula of resveratrol was obtained **A**, and the structure of C_14_H_12_O_3_ was optimized to obtain the most stable molecular conformation **B**. **C** Five sites (a-e)were selected as the initial positions for to calculate the adsorption energy of Ca on C_14_H_12_O_3_. **D** The side view and top view of (a) the initial adsorption position of Ca on the geometric structure of C_14_H_12_O_3_ and (b) the equilibrium adsorption position of Ca on the geometric structure of C_14_H_12_O_3_. **E** The side view (a) and top view of (b) of electronic structure of Ca adsorbed on C_14_H_12_O_3_ surface. Yellow/blue regions indicate charge aggregation/depletion, respectively, and the isosurface measures 0.0013 e/Å3. **F** The side view (a) and top view of (b) of the equilibrium adsorption position of Ca on the geometric structure of oxalic acid (C_2_H_2_O_4_)

The molecular arrangement of resveratrol was initially determined (Fig. 12A) and subsequently optimized to attain its most stable conformation (Fig. 12B). Informed by the molecular structure of resveratrol (Fig. 12C), we selected five preliminary positions for calculating the adsorption of Ca on C_14_H_12_O_3_. Our computational analysis revealed negative adsorption energies for all initial arrangements. Notably, the configuration where Ca was positioned directly above the center of the 3,5-hydroxybenzene ring exhibited the minimum energy (− 0.874 eV) and the closest adsorption distance (2.39 Å, as presented in Table 2) (Fig. 12D). Consequently, the adsorption of Ca induced changes in the geometric structure of C_14_H_12_O_3_, leading to charge accumulation between Ca and the carbon atoms of C_14_H_12_O_3_. A Bader charge assessment demonstrated the transfer of 0.855 e− charges from Ca to C_14_H_12_O_3_ during the adsorption event (Fig. 12E). Lastly, to provide a comparative perspective, we examined the adsorption energy of oxalic acid in relation to Ca, revealing that the most stable configuration exhibited an adsorption energy of − -0.68 eV (Fig. 12F), a value greater than − 0.874 eV.

**Table 2:** The adsorption energies of Ca in different adsorption positions on C_14_H_12_O_3_ surface

Adsorption site	a	b	c	d	e
Eads(ev)	− 0.874	− 0.359	− 0.341	− 0.443	− 0.439

## Discussion

The conditions of kidney stones and diabetes are widespread medical issues that greatly affect the wellbeing of those afflicted. While the relationship between these two conditions has been well established [33], and resveratrol has shown promising results as a potential treatment option in previous studies [8], additional inquiry is needed to attain a more thorough comprehension of their common pathogenic mechanisms and the therapeutic potential of resveratrol supplementation in both disorders. Given the substantial public health and economic burden, this study aims to screen for candidate diagnostic biomarkers for kidney stones in individuals with diabetes, and to evaluate the therapeutic potential of resveratrol, so as to provide optimal prevention, diagnosis and treatment strategies for these conditions.

The intersection of genes associated with kidney stones and diabetes has been the subject of ongoing research in the medical field [34]. The results of the study presented here open up new avenues for exploring the underlying mechanisms of these two conditions and the potential for developing targeted therapies. Through the use of advanced analytical methods, including WGCNA and DEGs, the study was able to identify a total of 11 potential target genes associated with both diseases, and the expression levels of these identified genes exhibited a positive correlation. Functional analysis using GO, KEGG, and Reactome revealed a number of biological processes and pathways that were enriched to ascertain potential modes of action. The outcomes of GO analysis indicated that the genetic makeup were concentrated in collagen catabolic process, and extracellular matrix structural constituent, and collagen-containing extracellular matrix. The genes exhibited a significant enrichment in KEGG signaling pathways, particularly those related to fructose and mannose metabolism, cell adhesion molecules, and ECM-receptor interaction. Reactome analysis further demonstrated a close association between the targets and extracellular matrix organization, collagen fibril assembly, and cell junction organization. Our findings indicate a correlation between the presence of shared genetic factors and the manifestation of organ interstitial fibrosis, a crucial impact on the pathogenesis of both nephrolithiasis and diabetes [35]. Thus, it is imperative to address the deleterious effects of elevated glucose levels and kidney stones on organ fibrosis and to explore the possibility of targeting these genetic determinants as a promising therapeutic strategy for individuals suffering from both disorders.

The utilization of multiple algorithms, such as LASSO regression, RF, and SVM-RFE, allowed for a comprehensive assessment of a set of 11 candidate biomarkers. Upon comparison of the results from these algorithms, a single potential biomarker, IL11, was identified as a shared biomarker for both kidney stones and diabetes. Subsequent analysis of the expression profiles of IL11 in a patient cohort revealed that its expression levels were markedly raised in both the kidney stones and diabetic groups as compared to the control group. This was confirmed by analysis of three independent datasets, indicating that IL11 could potentially serve as a diagnostic biomarker for kidney stones in diabetic patients. The ROC analysis further supported this potential, with the highest accuracy achieved in different datasets. Additionally, subgroup analysis of IL11 expression levels based on tissue of origin showed that its expression was considerably upregulated in the "Calculi" cohort contrasted with the "Normal" cohort, however, no discernible disparity was detected between the cohorts labeled as "Normal" and "Control".

The IL-11 was first recognized in 1990 as a protein factor that promoted a murine plasmacytoma cell line, initially assumed to be dependent on IL-6 [36].The pleiotropic nature of IL-11 soon became apparent, as it was also characterized as a factor emanating from a bone marrow-derived cell line culture that impeded adipogenesis in preadipocytes [37]. Despite a surge of research on IL-11 during the 1990s, activity in this area has since diminished. Nonetheless, in the last decade, curiosity in IL-11 has flourished once again due to its involvement in numerous illnesses including diabetes and various kidney diseases [38–40]. The primary pathological process underlying type 2 diabetes mellitus (T2DM) is islet dysfunction, which is characterized by impaired insulin secretion [41]. In advanced T2DM patients, the abnormal buildup of ECM, known as fibrosis, is frequently observed and may contribute to organ malfunction [41]. Research involving bioinformatics analysis and animal models has revealed that high glucose levels could trigger the upregulation of IL11, leading to inflammation and fibrosis in islets, thus contributing to the development of islet dysfunction in T2DM [42]. Similarly, renal fibrosis is a common occurrence in various kidney diseases, which can lead to mechanical and electrical dysfunction and the progression of kidney dysfunction [40]. During the early stages of kidney stone formation or crystal-induced kidney injury, renal tubular epithelial cells (TECs) undergo epithelial-to-mesenchymal transition (EMT), contributing to renal fibrosis [43]. Findings indicate that the EMT of TECs is crucially reliant on IL-11, as TECs express IL-11 receptor-α (IL-11RA) and secrete IL-11 in reaction to injury in vivo or provocation by various pathological mediators in vitro, suggesting that this widespread cellular process can be triggered in response to damage stemming from diverse origins [38]. Prior research employing integrated imaging-genomics analyses on primary human fibroblasts has demonstrated that the principal transcriptional reaction to exposure to TGFβ1 is the elevation of IL11, which is essential for its pro-fibrotic impact [38]. In murine models, the manifestation of IL-11 transgenes or IL-11 administration precipitates kidney fibrosis or organ failure, whereas conditional elimination of Il11RA1 in TECs diminishes pathogenic signaling, tissue injury, and offers protection against disease [40]. Furthermore, anti-IL11 treatment correlates with a decrease in renal pathology and enhanced renal function, without eliciting the side effects observed with anti-TGFβ, such as augmented tubule damage or renal inflammation [40]. These insights emphasize the crucial involvement of IL-11 signaling in TECs during impeded kidney regeneration and propose the potential of IL-11 as a therapeutic target for initiating intrinsic healing processes in both chronic and acute renal disorders.

A cohort investigation involving Korean adults devoid of nephrolithiasis at the onset found that males exhibited a heightened propensity for kidney stones development in correlation with elevated concentrations of glucose, HbA1c, and insulin resistance, encompassing the prediabetic spectrum [44]. Moreover, a positive association was discerned between HOMA-IR and the risk of nephrolithiasis in male subjects [45]. Contrarily, no substantial correlations were identified among female counterparts, suggesting the possibility of inherent gender disparities within this interrelation. Similarly, a prospective cohort study from Japan found that hyperinsulinemia was associated with kidney stones formation in men, but no such relationship was observed in women [46]. In this current research, IL11 expression was considerably increased in male patients but not in female patients, highlighting the potential sex-specific effects of IL11 in the diagnosis and treatment of kidney stones in diabetic patients. Influential factors in renal resilience to injury induced by nephrolithiasis, diabetes, and various pathologies impacting renal performance, include sex hormones [47]. Estradiol exerts a renoprotective capacity and mitigates fibrosis, whereas testosterone elicits a contrary outcome [48, 49]. Such disparities could potentially stem from the modulation of IL11 on TGFβ1 and collagen expression, subsequently altering in synthesis and extracellular matrix degradation. However, the precise role of IL11 in kidney stone formation and how sex differences in IL11 affect kidney stones development require further research for confirmation. Further investigation is necessary to validate both the exact involvement of IL11 in the formation of kidney stones, as well as to determine the manner in which sex-related variances in IL11 impact the development of kidney stones.

In this study, xCell enrichment analysis revealed a robust positive association between IL11 and various types of endothelial cells. Further exploration using the Nephroseq database showed that IL11 was notably elevated in the tubulointerstitium in contrast to the glomeruli, and it was predominantly over-expressed in papillary tips when compared to other structures in the kidney. Through a single-cell analysis in the HPA database, it was determined that IL11 was mainly enriched in kidney collecting duct cells and proximal tubular cells. Scholars widely accept Randall's plaque theory as the mechanism of stone formation, which suggests that crystal particle retention in the renal tubules is a prerequisite for the formation of calcified plaques [50]. The deposited crystal particles can lead to mechanical injury or inflammatory immune response, causing damage to the renal tubules, promoting further crystal deposition and ultimately forming renal papillary calcified plaques [51]. The process of crystal deposition can cause kidney damage and renal fibrosis, in which IL11 and its receptor may play a crucial role. These findings shed light on the expression and distribution of IL11 in the kidney, providing valuable insights for further investigation into its role in renal biology. Notably, it was discovered that over-expression of IL11 is associated with chronic kidney disease and correlates positively with low GFR and elevated serum creatine levels, indicating that IL11 could potentially have a detrimental impact on kidney function.

As per the median expression of IL11, the male RP papillary tissues were bifurcated into two subgroups. Upon conducting subgroup analyses, the GSVA analysis revealed that several biological functions and pathways linked with renal fibrosis, such as extracellular matrix and basement membrane, collagen fibril formation and organization, epithelial mesenchymal transition, and focal adhesion, were enriched. Prior studies have demonstrated that the expression of the epithelial cell marker, calbindin-E, gradually decreases during the process of EMT in TECs, while new mesenchymal cell characteristics like α-SMA and vimentin are acquired, and components of the interstitial matrix like fibronectin and type I collagen are produced [52–55]. IL11 plays a critical role among the various factors regulating EMT in different ways [56]. Atypical accumulation of ECM constituents, such as fibronectin and type I collagen, characterizes renal fibrosis [57, 58]. The kidney encounters a plethora of pathological influences, encompassing injury, infection, inflammation, hemodynamic disturbances, and immunological responses, which inflict harm upon intrinsic cellular components and culminate in substantial collagen deposition [59–61]. Consequently, an escalating progression of renal parenchymal fibrosis, fibrotic scarring, and eventual deterioration of renal functionality ensues, as corroborated by our research findings.

As revealed by the GO analysis between subgroups, DEGs were found to be enriched in ECM organization, collagen binding, and collagen-containing ECM, while KEGG analysis revealed enrichment in glycolysis and gluconeogenesis, PI3K-Akt signaling, endocrine, and calcium reabsorption pathways. Fibrosis constitutes a pathological phenomenon distinguished by the excessive accumulation of collagen-enriched ECM, resulting in the gradual substitution of functional parenchymal tissue and impaired organ performance [62]. Renal fibrosis serves as a defining feature of end-stage chronic kidney disease, afflicting approximately 20 million adults in the USA and roughly 10% of the global populace [63–65]. Persistent ECM deposition and anomalous fibroblast activity disrupt kidney structure, thereby compromising the functionality of the vasculature, glomeruli, and tubule-interstitium, and subsequently diminishing blood flow and overall organ performance [66, 67]. The release of IL-11 by fibroblasts and epithelial cells plays a pivotal role in fibroblast stimulation, myofibroblast differentiation, and extracellular matrix deposition, facilitated by both Smad-independent mechanisms and the PI3K/Akt pathway [68, 69].Thus, targeting these fibrotic mediators or signaling pathways may represent a promising therapeutic strategy for combating fibrotic diseases, including kidney stones associated with diabetes. In addition, the infiltration of immune cells into the tubulointerstitium following injury is known to contribute significantly to the progression of various kidney diseases [70, 71]. According to the GSVA immune analysis, patients classified as IL11-high subgroup exhibited notably elevated levels of NKT cells. Interestingly, the expression of IL-11 exhibit a positive association with the levels of NKT cells. NKT cells constitute a unique lymphocyte population expressing both NK receptors and TCRs, and secrete various cytokines that exert regulatory functions. NKT activation has been demonstrated to contribute to collagen deposition and myofibroblast formation, and is closely associated with the extent of tubulointerstitial fibrosis and subsequent decline of renal function, indicating a direct connection between the presence of NKT cells in the tissue and fibrosis, particularly in cases of obstructive nephropathy, such as kidney stones [72]. Taken together, these findings furnish a thorough understanding of the functional disparities between the two subtypes and their potential implications for future studies. They also highlight the importance of targeting fibrotic mediators and immune cells as potential therapeutic strategies for treating fibrotic diseases, including kidney stones associated with diabetes.

The present study utilized Mfuzz to analyze the expression of IL11, followed by clustering into 100 groups. By comparing kidney stones groups with normal groups, Cluster 9 emerged as the most strongly associated with IL11. Further investigation revealed that PROM1, TFPI2, and PFKFB3 are crucial genes involved in the regulation of IL11 expression in patients with kidney stones and diabetes. To better understand the regulatory mechanisms underlying IL11, PROM1, TFPI2, and PFKFB3, a comprehensive network was constructed that included miRNAs and IL11-related genes. In addition, a network of potential biomarkers and TFs was predicted. The results revealed 19 miRNAs and 9 TFs that are critical in this regulatory process. PROM1, also known as CD133, is expressed by renal progenitors and may serve as a marker of renal regeneration [73]. Targeting CD133 may be useful in inhibiting proliferation or inducing differentiation in renal nonmalignant pathology [74]. TFPI2, a Kunitz-type serine proteinase inhibitor family member primarily produced by endothelial cells originating from various blood vessels, contributes to diabetic nephropathy progression by facilitating renal fibrosis and EMT through the modulation of the SMURF2/SMAD7-mediated TGF-β/Smad signaling pathway [75]. In recent years, multiple studies have suggested that PFKFB3 is a central modulator in metabolic reprogramming. PFKFB3 expression was upregulated in endothelial cells exposed to high glucose conditions and contributed to diabetic kidney disease by enhancing ECM production by interacting with TGF-β [76, 77].

In the present investigation, the capacity of resveratrol, a natural polyphenolic compound, to interact with IL11 was evaluated, revealing a potent binding affinity. This interaction implies that resveratrol holds promise as a therapeutic remedy for the management of diabetes and kidney stone conditions by targeting IL11. Resveratrol can be found in various plants such as Cassiae Semen and Polygoni Cuspidati Rhizoma Et Radix, as well as in commonly consumed dietary sources including grapes and peanuts, which have been utilized in traditional medicinal practices. The efficacy of resveratrol has been well-documented in treating a broad spectrum of ailments, encompassing kidney stones and diabetes [10, 78]. Its therapeutic effects have been attributed to its capacity to impede the process of tubular epithelial cell EMT, fibroblast proliferation and differentiation, as well as to hinder the activation of myofibroblasts and to ameliorate renal fibrosis in kidney stones and diabetes by obstructing the activity of proliferation-related signaling pathways of both epithelial and interstitial cells [11]. These findings validate the outcomes of our current research.

The development of drugs for the treatment of kidney stones is confronted with various challenges, including a lack of precise analytical tools. However, the emergence of supercomputers and software has opened up new avenues for investigating the interactions between atoms and molecules at the microscopic level. Unlike conventional experiments, computer simulations and calculations can provide a more detailed mechanism of action with greater accuracy and reliability [79]. This study employed first-principles calculations to scrutinize the basic issues related to Ca adsorption on the C_14_H_12_O_3_ surface, including the adsorption position, mode, and energy, to elaborate on the process in which resveratrol restrains the creation of calcium oxalate stones at a structural level. The computational results showed that the binding of resveratrol to Ca is stable, with negative adsorption energies in all Ca/C_14_H_12_O_3_ initial configurations, indicating a strong adsorption effect of resveratrol on Ca. We defined the adsorption distance as the shortest distance from Ca to a specific atom on C_14_H_12_O_3_ after adsorption. Configuration (a) had the smallest adsorption energy and the most stable adsorption system, with Ca moving from the center of the 3,5-dihydroxyphenyl ring to a position diagonally above carbon 1. The relaxation and reconstruction of the C_14_H_12_O_3_ geometric structure demonstrated the strong interaction between Ca and C_14_H_12_O_3_. The charge transfer shown in the charge density difference map of the adsorption system indicated the stability of the adsorption between Ca and C_14_H_12_O_3_ in terms of electronic structure. By comparing the adsorption energy of Ca on the C_14_H_12_O_3_ surface with the C_2_H_2_O_4_ surface, the study concluded that the adsorption of resveratrol on Ca is stronger than that of oxalic acid, providing evidence for the development of drugs based on resveratrol to prevent and treat kidney stones.

Limitations are inherent in this study, despite including some appropriate datasets from the GEO database. The amount of data obtained remains restricted. Furthermore, the validation of the hub gene's diagnostic potential in kidney stones was limited to a solitary suitable dataset, necessitating additional multicenter external datasets to investigate the exact molecular mechanism underlying the hub gene. In addition, further biological experiments research and clinical trials are required to assess the correlation linking clinical parameters and the hub gene. Moreover, elucidating the underlying roles of IL11 in kidney stones with diabetes necessitates additional investigations. It is also imperative to clarify the precise mode of action of resveratrol in managing kidney stones with diabetes. In light of the constraints inherent in this investigation, the current research endeavors to furnish a preliminary outlook on the common biomarkers linked to both kidney stones and diabetes, as well as a significant theoretical and empirical basis for resveratrol in the treatment of these conditions.

## Conclusions

**Fig. 13:**
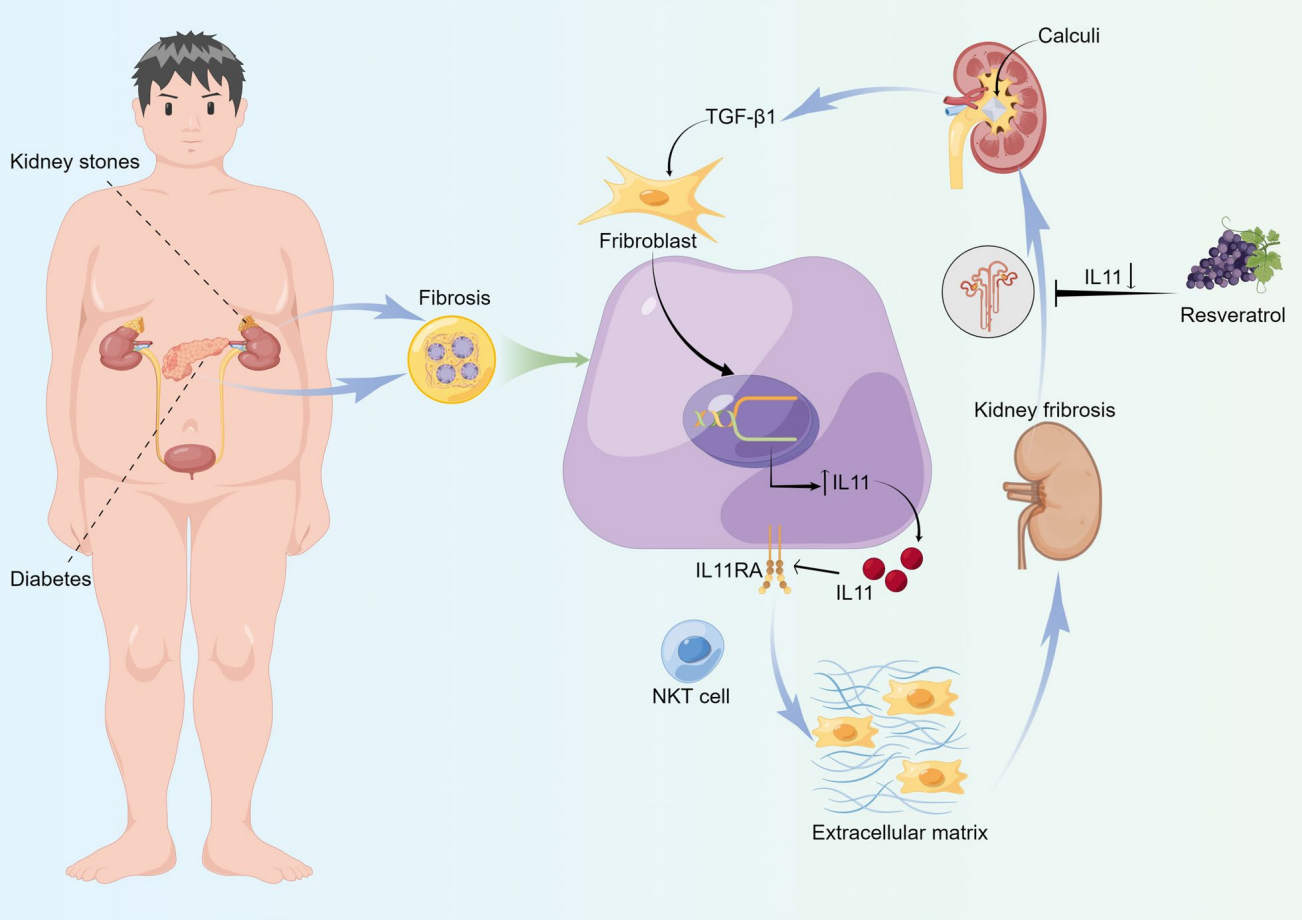
The pathogenic mechanisms shared between kidney stones and diabetes and the role of resveratrol

Our study constituted the initial identification of unique biomarkers and pathways shared between kidney stones and diabetes at the transcriptional level. Through the amalgamation of diverse datasets and bioinformatic techniques, we pinpointed IL11 as a core biomarker in the mutual mechanism underlying these conditions, thereby rendering it a promising therapeutic target (Fig. 13). Notably, our findings revealed that resveratrol exhibited a robust binding affinity for IL11, underscoring its potential as a therapeutic agent. Moreover, we harnessed supercomputing and quantum mechanical computational theories to scrutinize the preventive mechanism of resveratrol against calcium oxalate stone formation from an innovative and microscopic standpoint. Taken together, our research illuminated the intricate association between kidney stones and diabetes, paving the way for the creation of novel therapeutic approaches. Nonetheless, further research is warranted to enhance our understanding of IL11 targeting mechanisms and address any limitations in the study.

## Supplementary Information

**Additional file 1: Figure S1** WGCNA for GSE73680 and GSE41762. Hierarchical clustering tree of gene expression patterns in the GSE73680 dataset of kidney stones **A** and the GSE41762 dataset of diabetic** B**.

**Additional file 2: Figure S2** IL11 expression patterns identified by MFuzz.

## Data Availability

The datasets supporting the conclusions of this article are available in the GEO repository, including GSE73680, GSE41762 and GSE38642.

## Abbreviations

GEO: Gene Expression Omnibus Database
RP: Randall’s plaque
WGCNA: Weighted gene co-expression network analysis
TOM: Topological overlap matrix
GS: Gene significance
MM: Module membership
DEGs: Differentially expressed genes
FC: Fold change
PPI: Protein–protein interaction
GO: Gene ontology
KEGG: Kyoto Encyclopedia of Genes and Genomes
BP: Biological processes
MF: Molecular functions
CC: Cellular components
LASSO: Least absolute shrinkage and selection operator
RF: Random forest
SVM-RFE: Support vector machines recursive feature elimination
ROC: Receiver operating characteristic curve
AUC: Area under the curve
CI: Confidence interval
GSVA: Gene set variation analysis
ssGSEA: Single sample gene set enrichment analysis
TFs: Transcription factors
PDB: Protein Data Bank
IL11: Interleukin 11
Ca: Calcium
C_14_H_12_O_3_: Resveratrol
C_2_H_2_O_4_: Oxalic acid
DFT: Density functional theory
VASP: Vienna Ab-initio simulation package
GGA: Generalized gradient approximation
PAW: Projector augmented wave
ECM: Extracellular matrix
T2DM: Type 2 diabetes mellitus
TECs: Renal tubular epithelial cells
EMT: Epithelial-to-mesenchymal transition
NKT Cell: Natural Killer T Cell
