# Supplementary figures and images for: Elucidating shared biomarkers and pathways in kidney stones and diabetes: insights into novel therapeutic targets and the role of resveratrol
Source: J Transl Med. 2023 Jul 21;21:491. doi: 10.1186/s12967-023-04356-4 (PMC10360253; doi:10.1186/s12967-023-04356-4)

A

Sample dendrogram and trait heatmap

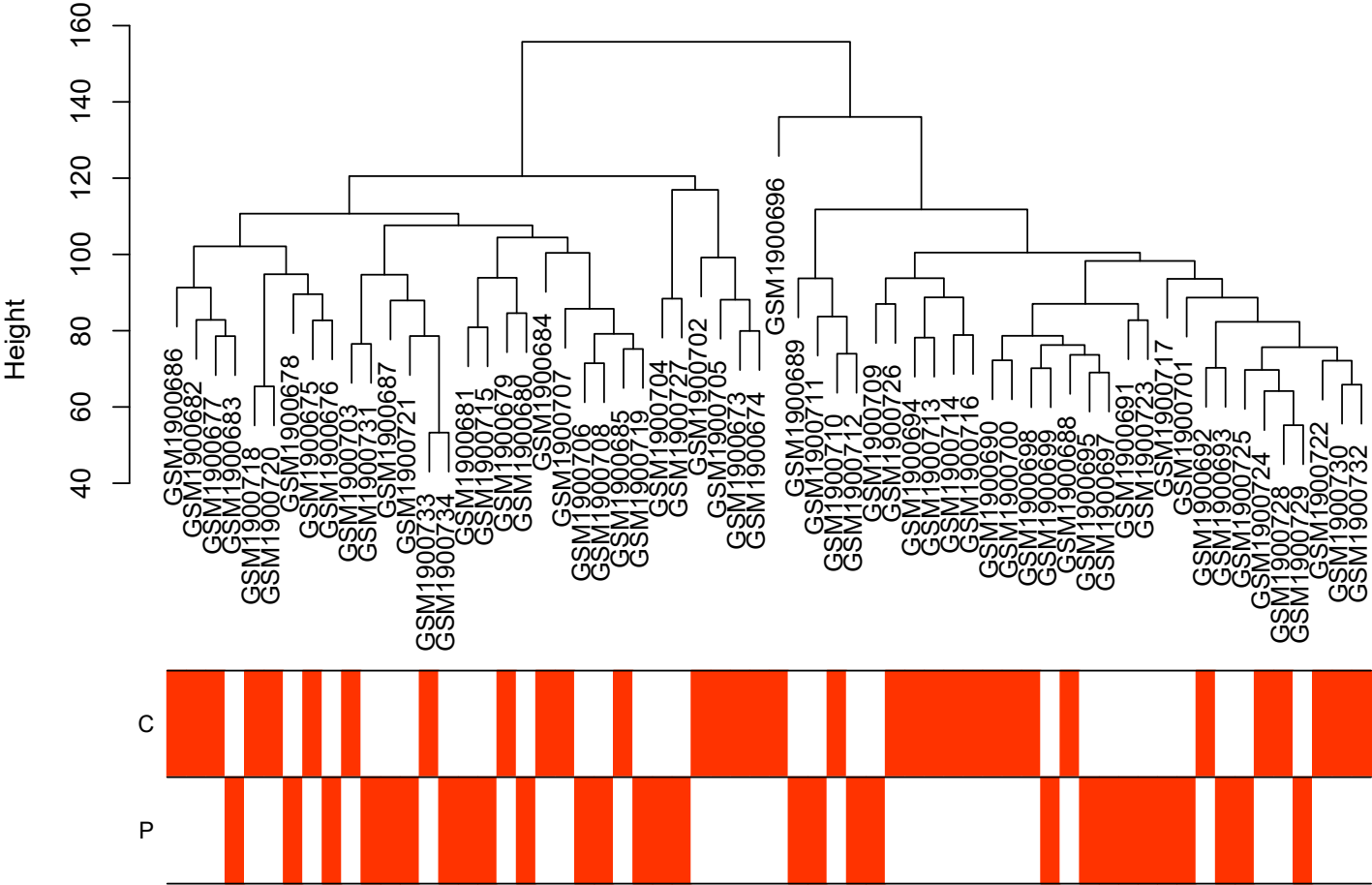

B

Sample dendrogram and trait heatmap

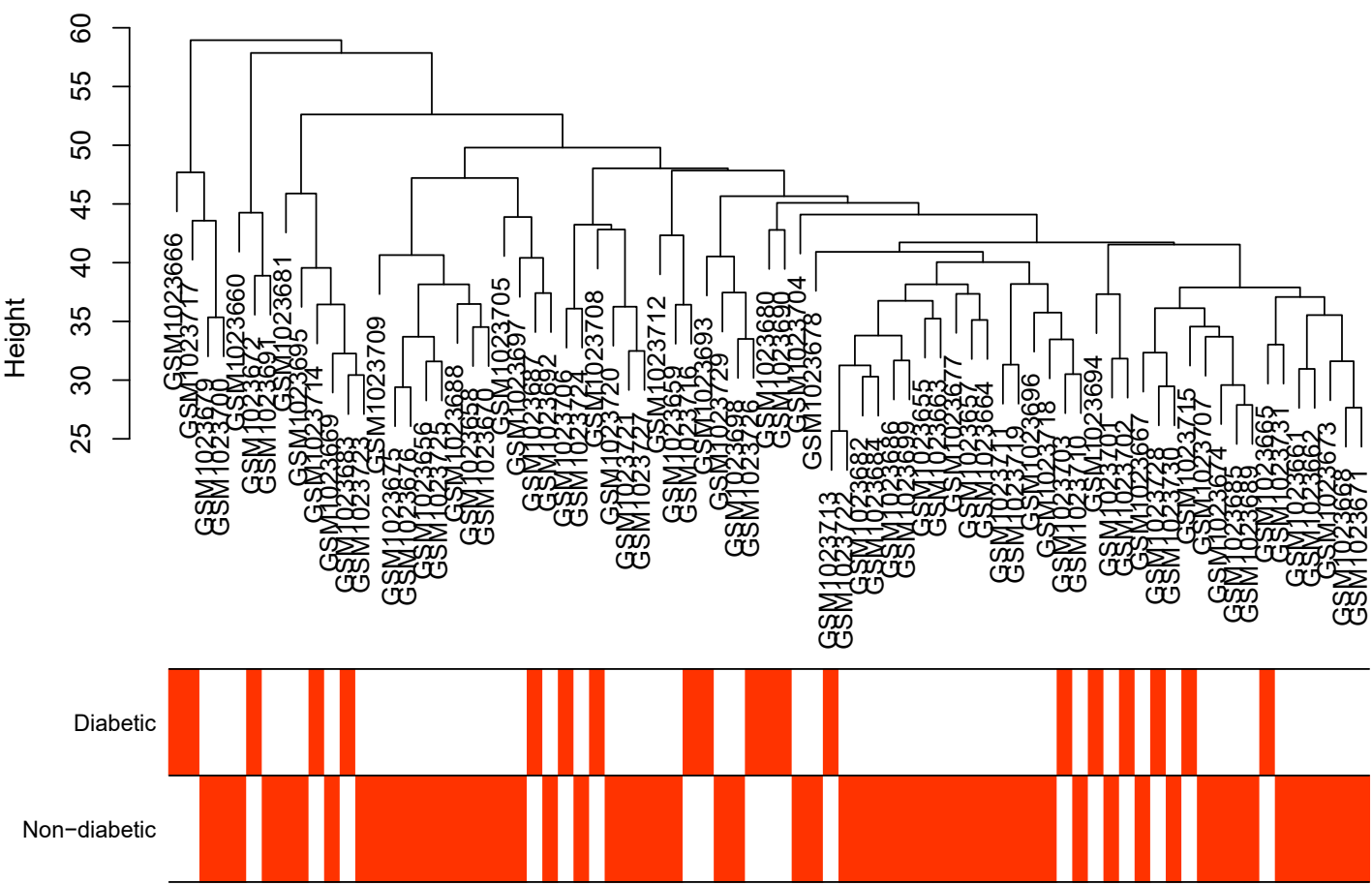

Supplement: Supplementary file 1 — Additional file 1: Figure S1 WGCNA for GSE73680 and GSE41762. Hierarchical clustering tree of gene expression patterns in the GSE73680 dataset of kidney stones A and the GSE41762 dataset of diabetic B. [file 12967_2023_4356_MOESM1_ESM.pdf]

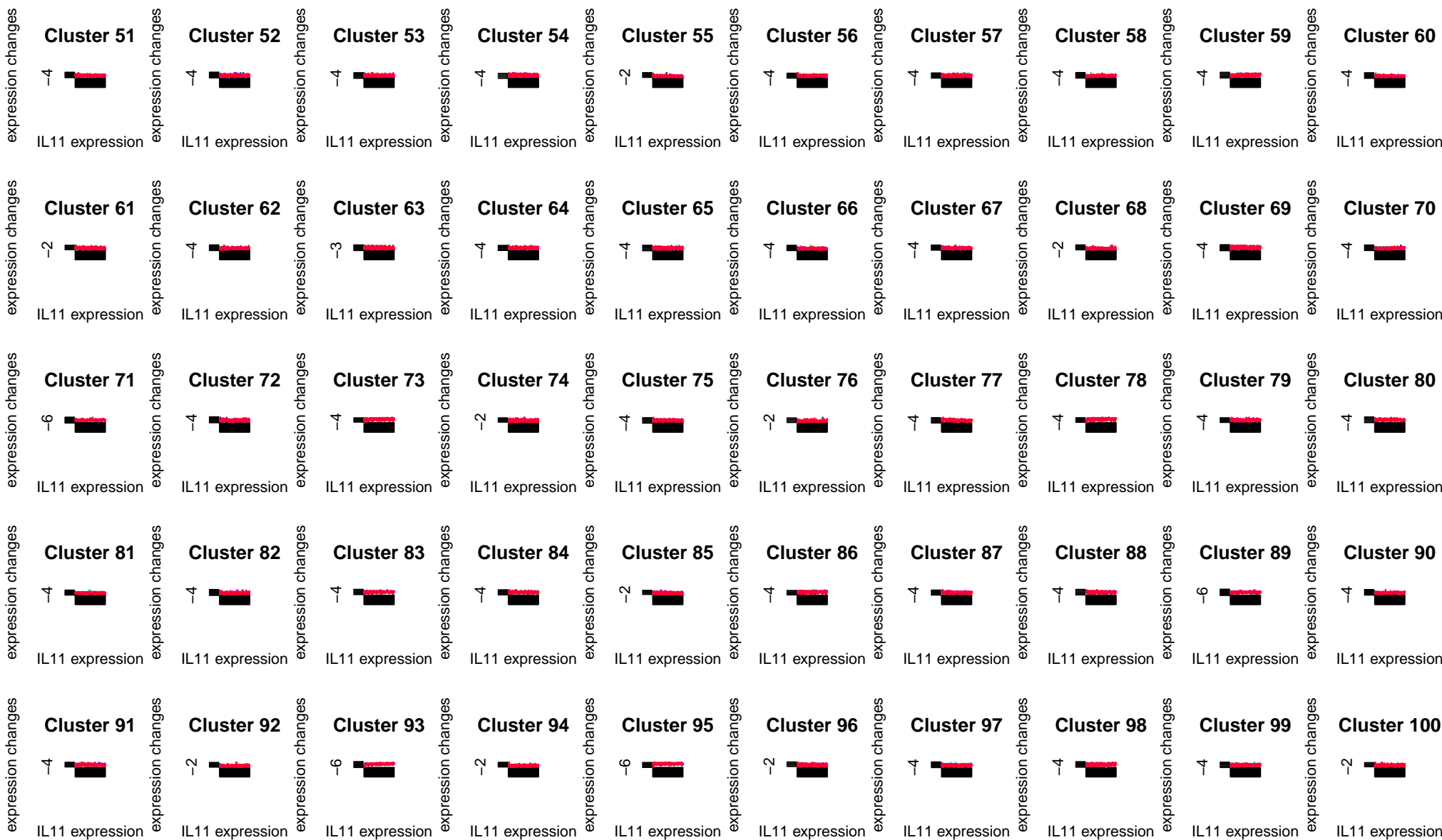

Supplement: Supplementary file 2 — Additional file 2: Figure S2 IL11 expression patterns identified by MFuzz. [file 12967_2023_4356_MOESM2_ESM.pdf]
